# Comparative Analysis of Salivary Tumor Marker CA-125 Among Oral Squamous Cell Carcinoma Patients and Healthy Individuals

**DOI:** 10.3390/dj13050194

**Published:** 2025-04-29

**Authors:** Riham Mohammed, Mariam El Sheikh, Nada Tawfig Hashim, Nallan CSK Chaitanya, Ahmed Suleiman

**Affiliations:** 1 RAK College of Dental Sciences, RAK Medical and Health Sciences University, Ras-AlKhaimah 12973, United Arab Emirates; nada.tawfig@rakmhsu.ac.ae (N.T.H.); krishna.chytanya@rakmhsu.ac.ae (N.C.C.); 2 Faculty of Dentistry, University of Khartoum, Khartoum 11115, Sudan; mariomaa79@gmail.com (M.E.S.); newsulaiman@yahoo.com (A.S.)

**Keywords:** oral cancer, OSCC, saliva, biomarkers, CA-125

## Abstract

**Background/Objectives**: In Sudan, oral cancer is one of the top ten most common cancers, with OSCC representing the majority of the cases. To date, despite the fact that saliva can be collected simply and non-invasively, there is no approved salivary tumor marker for OSCC. This study aimed to investigate the reliability of salivary CA-125 as a tumor marker for OSCC by measuring and comparing its level among OSCC and healthy individuals as well as its level across different histopathological grades. **Methods**: A total of 100 subjects were enrolled; 50 were patients with OSCC, while the other 50 were matched healthy individuals. Non-stimulated whole saliva was collected before the administration of definitive treatment, and the concentration of salivary CA-125 was quantified using an automated immunoassay analyzer that employs a one-step sandwich fluorescent enzyme immunoassay (FEIA). **Results**: The level of salivary CA-125 was 342.65 U/mL in the cases group, which was significantly increased compared with 203.65 U/mL in the healthy controls (*p* = 0.017). Statistically significant differences in the level of salivary CA-125 among different histopathological grades were observed (*p* = 0.014). The sensitivity, specificity, accuracy, and positive and negative predictive values were 48%, 78%, 63%, 68.6%, and 60%, respectively. **Conclusions**: This study suggests that salivary CA-125 could serve as a potential tumor marker for OSCC. However, its clinical application requires further validation.

## 1. Introduction

Oral cancer (OC) remains a significant global public health concern [1] and continues to rank among the top ten causes of cancer-related mortality in the United States [2]. According to the International Classification of Diseases and Related Health Problems (ICD-11), oral cancer encompasses all malignancies originating from the lips, oral cavity, and pharynx [3]. In Sudan, OC is one of the most prevalent malignancies, primarily due to the widespread use of Toombak, a form of smokeless tobacco commonly dipped in the oral mucosa [4]. Despite its high incidence, OC is not listed among the top ten causes of death in the country [5].

Oral squamous cell carcinoma (OSCC), which accounts for approximately 90% of all oral cancers, has a favorable prognosis, with an 80–90% survival rate when detected early. Nevertheless, the World Health Organization reports that oral cancer remains one of the malignancies with the highest fatality rates, exhibiting a five-year mortality rate of 45–50% [1,2,6,7,8]. This rate has remained largely unchanged over the past five decades, despite major advancements in cancer therapeutics [1,2,7]. Additionally, OC is associated with substantial morbidity, often resulting in functional impairment and facial disfigurement [8].

Tumor markers are biological substances produced by cancer cells or the body in response to malignancy and may be detected either within tissues or in circulating body fluids such as serum, saliva, urine, or cerebrospinal fluid [9,10]. Among these, blood and saliva are the most extensively studied fluids for the detection of OSCC-related tumor markers [2]. Compared with serum-based testing, saliva offers a non-invasive, safe, and cost-effective medium, making it a promising tool for early diagnosis, prognosis assessment, and post-treatment monitoring of OC [1,11].

Cancer antigen 125 (CA-125), also known as MUC16, is a high-molecular-weight glycoprotein belonging to the mucin family [12]. It is shed from the surface of cancer cells into body fluids, including saliva and blood, and has been reported to be elevated in various epithelial malignancies such as ovarian, breast, and oral squamous cell carcinoma [7].

Currently, OSCC diagnosis primarily relies on clinical expertise and histopathological evaluation of suspicious lesions. However, these conventional methods may fail to detect malignancies in anatomically hidden or early-stage lesions. Therefore, identifying sensitive and specific salivary biomarkers such as CA-125 could significantly enhance screening strategies, particularly in high-risk populations [1,2].

This study aims to evaluate the reliability of salivary CA-125 as a potential tumor marker for OSCC by comparing its levels in OSCC patients and healthy controls and by investigating its association with different histopathological grades of the tumor.

## 2. Materials and Methods

This hospital-based comparative analytical cross-sectional study was conducted at Khartoum Teaching Dental Hospital over a period of 12 months. A total of 100 Sudanese participants were enrolled and evenly allocated into two groups: 50 patients who were newly diagnosed with oral squamous cell carcinoma (OSCC) and 50 healthy individuals serving as the control group.

### 2.1. Selection of Cases and Healthy Participants

Case definition: OSCC patients included in the study were those with histologically confirmed squamous cell carcinoma who had not received any form of treatment (surgery, radiotherapy, or chemotherapy) before enrollment. The diagnosis was established by an incisional biopsy and histopathological examination, including grading of tumor differentiation.

Control definition: Healthy individuals were defined as those free from OSCC, with no clinical signs or symptoms suggestive of oral cancer. They were recruited from various departments of the hospital and included companions and relatives of OSCC patients, helping minimize socioeconomic variability.

Inclusion criteria: Sudanese individuals aged 18–85 years were eligible for both groups.

Exclusion criteria:

- Refusal to participate
- History of premalignant oral lesions or other forms of oral cancer
- Past or current diagnosis of systemic malignancies known to elevate CA-125 levels (e.g., ovarian, breast, endometrial, or colorectal cancers)
- Oral mucosal conditions with bleeding potential (e.g., acute gingivitis, traumatic ulcers)
- Xerostomia or salivary gland disorders affecting saliva quantity or quality
- Inability to provide a minimum of 3 mL of saliva
- Pregnant, lactating, or menstruating women (to control for hormonal variation in CA-125 levels)

**Figure 1:**
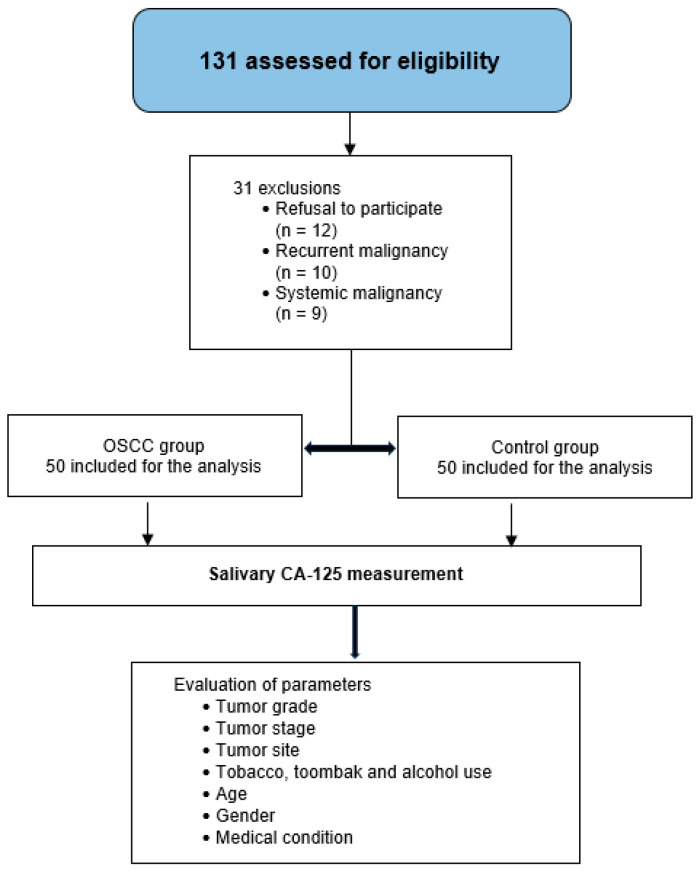
Study flowchart showing participant selection, exclusion criteria, group allocation, and variables analyzed.

The flow of participant recruitment, eligibility screening, exclusions, group allocation, and assessed parameters is illustrated in Figure 1.

Data collection procedure: All participants signed informed consent forms. OSCC patients underwent comprehensive clinical, radiological, and histopathological evaluation, while control participants were assessed by history and clinical oral examination alone.

Each OSCC patient underwent a thorough assessment, beginning with a structured interview to obtain demographic data, personal habits—particularly the use of Toombak—past medical history, and tumor duration. This was followed by a comprehensive clinical examination, which involved evaluation of the primary tumor’s size (T), anatomical location (excluding tumors of the tonsils and pharyngeal wall), and assessment of cervical lymph node involvement (N). Radiographic imaging included contrast-enhanced computed tomography (CT) of the head and neck to evaluate local tumor extension and nodal status, while magnetic resonance imaging (MRI) was specifically used for tongue tumors. To assess distant metastasis (M), chest X-rays and abdominopelvic ultrasound scans were performed. In addition, dental panoramic tomography (DPT) was used to detect bone invasion or involvement of tooth sockets. Based on these clinical and radiological findings, tumors were staged according to the American Joint Committee on Cancer (AJCC) TNM classification system. Subsequently, histopathological grading of OSCC was conducted using Broder’s classification system [13], which categorizes tumors based on the degree of cellular differentiation and keratinization into well-differentiated (G1), moderately differentiated (G2), and poorly differentiated (G3) carcinomas.

Grading in Multi-Site OSCC Cases: Approximately 30% of the OSCC cases included in this study involved tumors affecting multiple anatomical sites. In these cases, histopathological grading was determined based on the most poorly differentiated site identified among the biopsy specimens collected from each site. This decision was made in alignment with clinical oncological practice, where the least differentiated component is often considered the most prognostically significant. By grading based on the most aggressive area, we aimed to avoid underestimating tumor severity and its potential correlation with salivary CA-125 levels. This method ensures consistency and avoids the confounding influence of intra-tumoral heterogeneity, which is well documented in OSCC. We acknowledge that future studies could benefit from more advanced spatial mapping of tumor differentiation, but our approach remains clinically relevant and consistent with protocols used in histopathological reporting of multifocal disease.

### 2.2. Saliva Collection

Unstimulated whole saliva was collected using the passive drooling method. Samples were collected before biopsy procedures between 8:00 AM and 2:00 PM to minimize diurnal variation.

Participants were provided with standardized pre-collection instructions to ensure sample consistency and minimize contamination. They were asked to refrain from eating, drinking, chewing, brushing, or using mouthwash for at least one hour before saliva collection [7,8,14], and to rinse their mouths thoroughly with water at least five minutes before the procedure to eliminate debris and reduce bacterial load [15,16]. During the collection, participants were seated in an upright position and instructed to swallow once, tilt their heads slightly forward, and allow saliva to accumulate in the mouth before spitting into a sterile plastic container. This process continued for 5 to 15 min or until a minimum of 3 mL of unstimulated whole saliva was obtained.

**Figure 2:**
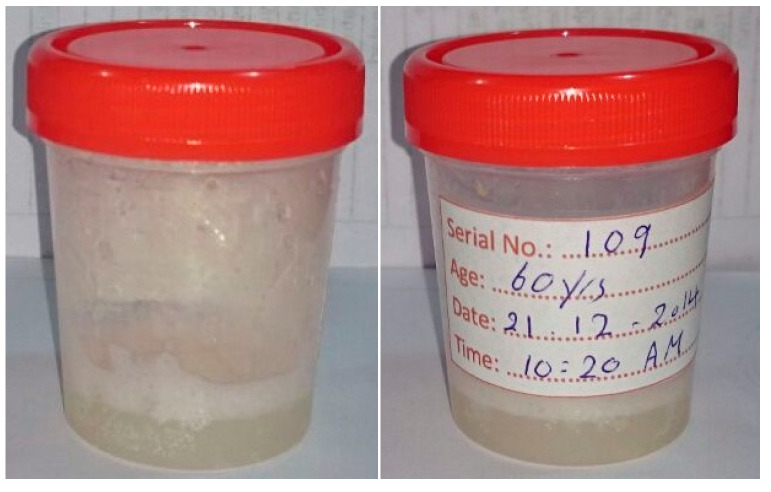
Saliva sample. Collection of unstimulated whole saliva in sterile, labeled containers under standardized conditions ensured proper identification and traceability of patient samples.

Samples were labeled with serial number, age, date, and time (Figure 2) and immediately transported to the laboratory for processing within a maximum of 3 h to maintain protein stability.

### 2.3. Quantification of Salivary CA-125

Salivary CA-125 levels were quantified using a validated automated immunoassay system (AIA-360, Tosoh, Tokyo, Japan), which employs a one-step sandwich and competitive fluorescent enzyme immunoassay (FEIA) technique with high sensitivity and specificity.

Saliva samples were processed under standardized conditions to preserve the stability of CA-125 levels and ensure reliable measurement throughout the analysis. A volume of 1 mL of each sample was transferred into a sterile conical centrifuge tube using a pre-calibrated 1000 μL air-displacement micropipette. An equal volume of sterile normal saline was then added to dilute the sample. The mixture was vortexed for 30 s to ensure homogeneity and subsequently centrifuged at 5000 rpm for 10 min to eliminate squamous cells and cellular debris. A 750 μL aliquot of the resulting clear supernatant was carefully pipetted into an assay cup using a fresh pipette tip for each sample to prevent cross-contamination. The assay cups, along with the reagent cartridges, were loaded into the AIA-360 automated immunoassay analyzer (Tosoh, Japan), which quantified salivary CA-125 levels using a one-step sandwich and competitive fluorescent enzyme immunoassay (FEIA) technique. The concentration of CA-125 was obtained within 20 min. The analyzer was calibrated at the start of the study, and recalibration was performed monthly using manufacturer-supplied standards. Strict quality control measures were adhered to throughout the study to ensure analytical accuracy and reliability.

**Figure 3:**
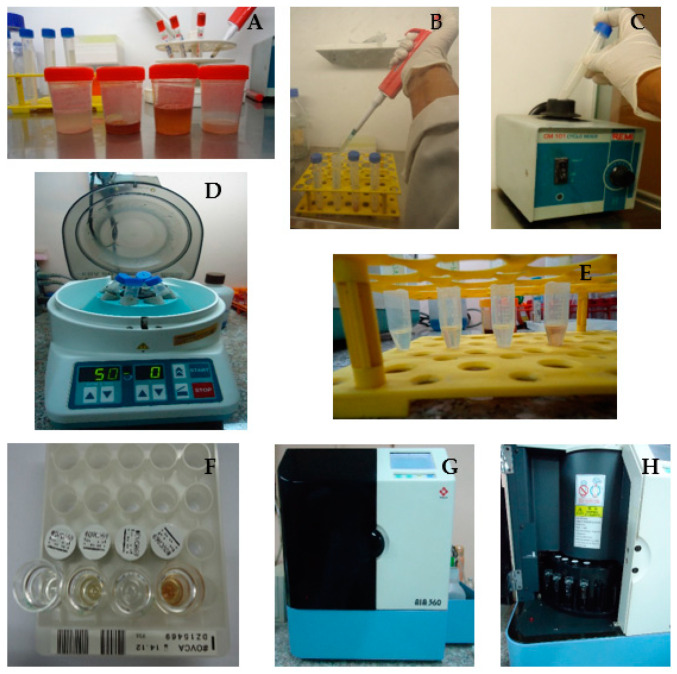
Stepwise procedure of salivary sample processing and biomarker analysis for CA-125 quantification. (**A**) Saliva samples. (**B**) Decontamination of the sample. (**C**) Sample vortexed using a CM 101 Cyclo Mixer, REM, India to ensure uniform mixing of the sample components and equal suspension of analytes prior to centrifugation or further biochemical analysis. (**D**) Centrifugation of salivary samples using EBA20 Centrifuge, Hettich Zentrifugen, Germany. This step separates the supernatant from cellular debris, enabling the collection of the clear fluid phase for biomarker analysis. (**E**) The resultant supernatant fluid. (**F**) Standard and OVCA reagent cups. (**G**) The Automated Enzyme Immunoassay Analyzer (AIA-360, Tosoh, Japan), which was used to quantify the salivary CA-125. (**H**) Loading saliva sample and OVCA reagent cups in the sample holder and reagent cup holder within the main carousel of the analyzer for the final biochemical analysis.

Photographic documentation of the salivary sample processing procedures is provided, illustrating adherence to standardized protocols for biomarker analysis, in Figure 3.

### 2.4. Statistical Analysis

Data were analyzed using SPSS version 21. Descriptive statistics (means, standard deviations, medians, frequencies) were used to summarize demographic, clinical, and biomarker data.

To check comparability of the two groups, an Independent t-test was used to compare the mean age between OSCC and control groups, while the chi-square test was used to analyze categorical variables such as sex distribution, tobacco habits, and other demographic variables.

The distribution of salivary CA-125 levels was assessed for normality using the Shapiro–Wilk test. Results indicated that the data were not normally distributed in both the OSCC group (*W* = 0.932, *p* = 0.006) and the control group (*W* = 0.890, *p* < 0.001). Therefore, non-parametric tests were appropriately used for group comparisons. (Mann–Whitney U test to compare salivary CA-125 levels between OSCC and control groups and between male and female participants in each group, and Kruskal–Wallis test to compare CA-125 levels across different histopathological grades, anatomical sites, and Toombak usage statuses).

A cut-off point for salivary CA-125 was defined at 354.40 U/mL, representing the upper bound of the 95% confidence interval of the control group mean (approximated by x + 2SE), used to distinguish positive from negative CA-125 results.

Spearman’s rho correlation was used to examine the association between salivary CA-125 levels and age within each group. A *p*-value < 0.05 was considered statistically significant for all tests.

## 3. Results

The total sample size consisted of 100 participants, with 50 patients diagnosed with OSCC and 50 matched healthy controls.

### 3.1. Participant and Variable Distribution

A comprehensive comparison between the OSCC and control groups is presented in Table 1. Both groups were matched for age and gender, with males comprising 68% and females 32% in each group. The mean age was comparable (OSCC: 59.72 ± 15.35 years; control: 58.26 ± 14.98 years; *p* = 0.631). In terms of Toombak use, 38% of OSCC patients were current users and 24% were ex-users, while 34% of the control group were current users and 24% ex-users. Non-users made up 38% and 42% of the OSCC and control groups, respectively (*p* = 0.900). Alcohol consumption and smoking habits were similarly distributed across both groups, with non-users comprising the majority.

The proportion of participants with chronic medical conditions—such as hypertension, type 2 diabetes mellitus, and controlled ischemic heart disease—was slightly higher in the OSCC group (18%, n = 9) compared with the control group (16%, n = 8), with no statistically significant difference (*p* = 0.940). These findings reflect the balanced distribution of baseline characteristics and comparability between the two groups (Table 1).

Regarding the histopathological grading of OSCC patients, the majority were diagnosed with well-differentiated squamous cell carcinoma (G1), accounting for 68% (n = 34). Moderately differentiated carcinoma (G2) was reported in 22% (n = 11), and poorly differentiated carcinoma (G3) in 10% (n = 5).

The distribution of TNM classification (tumor staging) among OSCC patients indicated a predominant presentation at advanced tumor stages. A substantial majority of patients (88%) were diagnosed at late TNM stages, with Stage IV being the most prevalent, observed in 86% of the cases (n = 43), and only 2% (n = 1) classified as Stage III. In contrast, early stages were significantly less frequent, with 6% of cases each reported at Stage I and Stage II (n = 3 each). This pattern suggests a notable delay in tumor detection, emphasizing the urgent need for enhanced screening and early diagnostic measures (Table 2).

The most commonly affected site in this OSCC cohort was the alveolar ridge, accounting for 32% (n = 16) of cases, followed by 30% (n = 15) of patients with tumors involving multiple oral or oropharyngeal sites. The least often involved anatomical locations were the anterior tongue and floor of the mouth, each representing 2% (n = 1) of the cases.

### 3.2. Comparative and Correlational Analyses

The median level of salivary CA-125 was significantly higher in the OSCC group, at 342.65 U/mL (range: 31.80–1340.00), compared with the control group with a median of 203.65 U/mL (range: 17.60–1011.20), showing statistical significance (*p* = 0.017).

**Figure 4:**
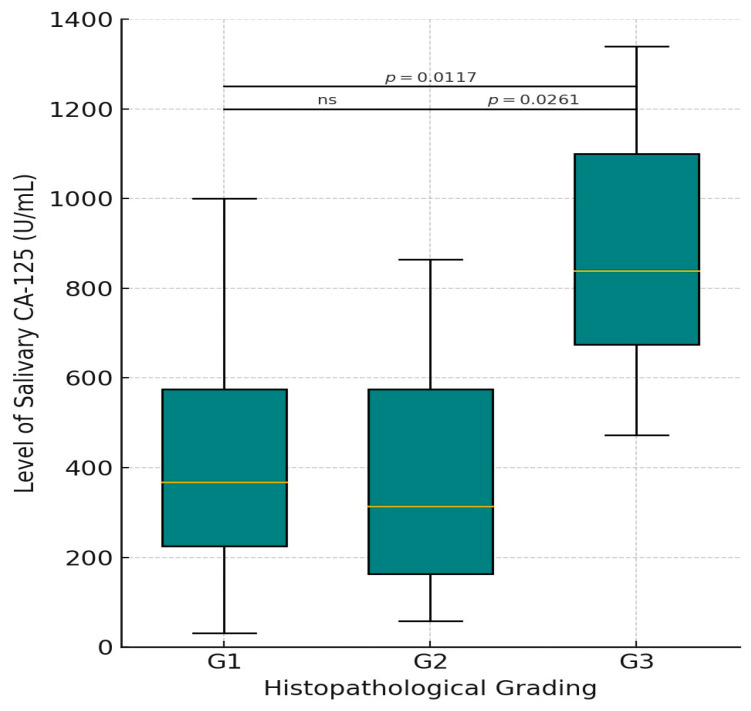
Box plot illustrating the distribution of salivary CA-125 levels (U/mL) across different histopathological grades of oral squamous cell carcinoma (OSCC). G1 = well-differentiated (n = 34), G2 = moderately differentiated (n = 11), and G3 = poorly differentiated (n = 5). The central horizontal line within each box represents the median; the box boundaries indicate the interquartile range (IQR), and the whiskers represent the minimum and maximum values within 1.5× IQR. ns: not significant. CA-125: cancer antigen 125.

The distribution of salivary CA-125 levels among patients with oral squamous cell carcinoma (OSCC) varied according to the histopathological tumor grade. The highest median CA-125 level was observed in the poorly differentiated tumors (G3), with a median of 839.40 U/mL (range: 472.40–1340.00), followed by the well-differentiated group (G1) at 336.50 U/mL (range: 31.80–1000.00), and the moderately differentiated group (G2) at 278.10 U/mL (range: 58.30–864.40). A statistically significant difference was observed between the groups (*p* = 0.014), suggesting that elevated salivary CA-125 levels may be associated with more aggressive histological features (Table 3) (Figure 4).

The Kruskal–Wallis test revealed a significant difference in salivary CA-125 levels among histopathological grades of OSCC (*p* = 0.014). Post hoc Mann–Whitney U tests with Bonferroni correction indicated significantly higher CA-125 levels in poorly differentiated OSCC (G3) compared with both well-differentiated (G1, *p* = 0.011) and moderately differentiated tumors (G2, *p* = 0.026). No significant difference was observed between G1 and G2 (*p* = 1.000) (Table 4).

The distribution of salivary CA-125 levels across various anatomical sites of OSCC is presented in Table 4. The highest median CA-125 level was observed in patients with tumors of the buccal mucosa (520.30 U/mL; range: 306.70–1340.00), followed by lesions on the lips (465.30 U/mL; range: 96.60–740.00), hard palate (410.20 U/mL; range: 155.10–513.00), and alveolar ridge (403.80 U/mL; range: 45.20–956.00). Lower median values were noted in tumors involving multiple sites (335.70 U/mL; range: 31.80–1000.00), the retromolar trigone (238.30 U/mL; range: 198.50–278.10), floor of the mouth (232.20 U/mL), anterior tongue (151.80 U/mL), and posterior tongue (91.90 U/mL; range: 58.30–125.50).

Although differences in median salivary CA-125 levels were observed among these anatomical locations, the variation did not reach statistical significance (*p* = 0.326). These findings suggest potential site-related variability in CA-125 expression, which warrants further investigation in larger sample sizes (Table 4).

The influence of gender on salivary CA-125 levels was assessed in both the OSCC and control groups, as shown in Table 5. In the OSCC group, the median salivary CA-125 level was higher in females (484.90 U/mL; range: 58.30–1340.00) compared with males (324.20 U/mL; range: 31.80–1000.00), although the difference was not statistically significant (*p* = 0.134). Similarly, in the control group, females exhibited a higher median CA-125 level (248.15 U/mL; range: 76.80–1011.20) than males (188.60 U/mL; range: 17.60–1000.00), with no significant difference noted (*p* = 0.167). These findings suggest a trend of higher CA-125 levels in females across both groups, though not reaching statistical significance (Table 5).

A very weak inverse relationship was noted between age and salivary CA-125 levels in the OSCC group, with a correlation coefficient of –0.046 (*p* = 0.750). In the control group, a weak positive linear correlation was observed (correlation coefficient = 0.257), which approached but did not reach statistical significance (*p* = 0.072).

Differences in salivary CA-125 levels were also examined based on Toombak use status (Table 6). In both groups, the highest median CA-125 levels were observed in non-snuff dippers. However, these differences were not statistically significant (*p* = 0.391 in the OSCC group and *p* = 0.628 in the control group).

### 3.3. Diagnostic Performance Evaluation

The diagnostic performance of salivary CA-125 in differentiating OSCC patients from healthy individuals is summarized in Table 7. Among the OSCC group, 24 participants tested true positive, while 26 tested false negative. In the control group, 11 individuals showed false-positive results, while 39 were correctly classified as true negative.

The calculated reliability indices of the test for salivary CA-125 were as follows: sensitivity = 48%, specificity = 78%, and overall accuracy = 63%. The positive predictive value (PPV) was 68.6%, while the negative predictive value (NPV) was 60%. These findings suggest a moderate discriminatory capacity of salivary CA-125 in detecting OSCC, supporting its potential as a supplementary biomarker pending further validation (Table 7).

## 4. Discussion

CA-125 was first introduced by Bast et al. [17] as a biomarker for ovarian cancer. Since then, its role has expanded, with evidence from Daoud and colleagues [18] showing that while CA-125 can be expressed under normal physiological conditions, its levels are markedly elevated in malignancies, as its expression is upregulated in malignant tumor cells and it is released into circulation. In ovarian cancer, serum CA-125 has been well-established as a diagnostic and prognostic tool, correlating strongly with tumor stage, differentiation, and nodal involvement [19,20,21]. Further advancement came from Di-Xia et al. [22], who demonstrated a significant association between CA-125 levels in saliva and serum, concluding that salivary CA-125 could offer better diagnostic accuracy.

Serum CA-125 was assessed as a tumor marker for OSCC before salivary CA-125, but it has shown limited value in detecting OSCC; this is supported by the studies of Zoller et al. [23] and Hoffmann et al. [24]. Salivary CA-125 has emerged as a promising alternative. Nagler et al. [8], Balan et al. [7], and Geng et al. [25] independently suggested its potential reliability for OSCC detection, prompting further investigation into its clinical utility.

In the present study, salivary CA-125 levels were found to be significantly elevated in OSCC patients compared with healthy individuals. This reinforces the findings of earlier studies and supports the hypothesis that CA-125, when measured in saliva, may reflect tumor-related changes in epithelial tissue and serve as a viable, non-invasive biomarker for OSCC detection. Most patients in this study were diagnosed with well-differentiated OSCC, contrasting with earlier studies such as those by Nagler et al. [8] and Geng et al. [25], where moderately differentiated OSCC was more common. These variations could be due to differences in population genetics, environmental exposures, and risk habits.

Unlike Geng et al. [25], who reported no significant relationship between salivary CA-125 levels and histopathological grades, the present study demonstrated a statistically supported association. Post hoc pairwise comparisons using the Mann–Whitney U test with Bonferroni correction revealed that CA-125 levels were significantly higher in poorly differentiated OSCC (G3) compared with both well-differentiated (G1, *p* = 0.0117) and moderately differentiated tumors (G2, *p* = 0.0261). No significant difference was observed between G1 and G2 (*p* = 1.000). These findings align with those of Ahmad et al. [26] and Younus et al. [27], who also reported an inverse relationship between tumor differentiation and CA-125 concentration. Interestingly, the median CA-125 level in well-differentiated OSCC was slightly higher than that in moderately differentiated cases. This unexpected trend may be attributed to sampling variability or limitations inherent in Broder’s classification system, which may lack the granularity provided by more contemporary histopathological grading frameworks.

Alveolar ridge involvement was the most frequently observed site of OSCC in this study, followed by tumors affecting multiple oral sites, and the least involved sites were the anterior tongue and the floor of the mouth. These findings are partly in agreement with other regional studies, such as that by Tarig et al. [28], though site distribution differs considerably across the literature. For example, Balan et al. [7] found the buccal mucosa to be the most commonly involved site, and the least involved sites were the floor of the mouth and alveolar ridge. On the other hand, Geng et al. [25] reported the tongue and gingiva as more frequently affected. while the palate was the least involved.

Such variations likely stem from different genetic backgrounds and carcinogenic exposures, such as the widespread use of Toombak in the Sudanese population, which may predispose individuals to lesions in the gingivolabial region and result in more extensive oral cavity involvement. Although no statistically significant association was found between tumor site and salivary CA-125 levels in the present study, this is consistent with the findings of Younus et al. [27]. In contrast, Ahmad et al. [26] did find a significant correlation, suggesting the need for further site-specific research.

The age profile of OSCC patients in this study closely mirrors findings from previous local and international studies. Similar to the observations by Geng et al. [25] and Balan et al. [7], no significant correlation was found between salivary CA-125 levels and age, indicating that age may not be a confounding factor in interpreting CA-125 levels. However, this contradicts the results reported by Younus et al. [27], who observed age-related differences in CA-125 levels, possibly due to demographic or methodological differences between the studies.

The gender distribution in this study reflected the commonly observed male predominance in OSCC cases, a trend attributed to the high prevalence of Toombak use, smoking, and alcohol consumption among Sudanese men. This observation is in line with the findings of Tarig et al. [28] and Geng et al. [25], though other studies, such as those by Nagler et al. [8] and Balan et al. [7], reported a higher proportion of female patients.

No significant difference in salivary CA-125 levels was observed between males and females in either group, supporting the findings of Balan et al. [7]. However, Younus et al. [27] reported higher CA-125 levels in males, possibly due to demographic differences and variations in exposure to risk factors.

To date, no published study has directly examined the relationship between Toombak use and salivary CA-125 levels. In the present study, higher CA-125 levels were noted among participants who did not use Toombak, though the difference was not statistically significant. These results suggest that while Toombak may play a role in OSCC pathogenesis, its influence on CA-125 expression remains unclear and warrants further exploration.

The challenge of defining a universal cut-off value for CA-125 lies in its variability among healthy individuals. The threshold identified in this study was closely aligned with that reported by Geng et al. [25], as both studies used identical methodologies and sample sizes to determine the upper bound of the 95% confidence interval. In contrast, other studies reported widely different cut-off values, likely due to variations in sample size, population characteristics, and calculation methods [7,8].

Diagnostic performance measures, including sensitivity, specificity, accuracy, and predictive values, were generally favorable in this study and comparable to the existing literature. However, sensitivity was lower, which may be explained by several factors. These include potential genetic differences in the Sudanese population; the predominance of Toombak-related OSCC with unique tumor biology, which may influence a difference in the biological behavior of the tumor as well as the expression of CA-125; and the use of FEIA instead of the more commonly used ELISA.

While FEIA offers automation and enhanced throughput, it may be susceptible to limitations such as cross-reactivity and the hook effect, which could compromise detection sensitivity [29,30,31]. Despite this, the technique remains a practical choice in clinical laboratories due to its high reproducibility and ease of use.

As McCauley et al. [32] note, a perfect diagnostic marker is rarely achieved in clinical settings. Acceptable levels of sensitivity and specificity are often determined based on disease characteristics and the intended use of the test. Screening tools, in particular, may prioritize sensitivity to reduce the risk of missed diagnoses.

According to Zhou et al. [33], the current study, along with most related research, is best classified within the exploratory phase of biomarker evaluation. Such studies are valuable for identifying promising markers but often rely on small samples and clear-cut cases, which can overstate diagnostic potential.

The biological plausibility of using CA-125 as a salivary biomarker in OSCC is reinforced by recent insights into the MUC16 gene, which encodes CA-125. MUC16 is a membrane-bound mucin expressed on epithelial surfaces, including the respiratory and reproductive tracts, and it is increasingly detected in head and neck squamous cell carcinomas [20].

In OSCC, dysregulated MUC16 expression has been linked to tumor progression, epithelial–mesenchymal transition, and immune evasion. Pro-inflammatory cytokines within the tumor microenvironment, such as IL-6 and TNF-α, may stimulate MUC16 transcription via JAK/STAT and NF-κB signaling pathways. Once cleaved from the epithelial membrane, the soluble extracellular domain of MUC16 (CA-125) can enter the salivary compartment either through local secretion or serum transudation via the gingival crevicular fluid, especially in the presence of mucosal disruption. These mechanisms offer a biological rationale for the elevated CA-125 levels observed in the saliva of OSCC patients [34,35,36].

In addition to CA-125, several other salivary biomarkers have been explored for OSCC detection. Interleukin-6 (IL-6), for example, shows elevated levels in OSCC but suffers from low specificity due to its role in systemic inflammation [37].

Cyfra 21-1, a cytokeratin 19 fragment, offers moderate diagnostic value but is not widely accessible in clinical laboratories. MMP-9 and CD44, both involved in tumor invasion and metastasis, have demonstrated varying degrees of utility. Compared with these, CA-125 benefits from the availability of standardized immunoassays and extensive characterization, although its use in oral cancer is still emerging. Our findings suggest that CA-125 could contribute meaningfully to a multi-marker salivary panel aimed at enhancing diagnostic sensitivity and specificity. Future studies should explore such combinations for improved accuracy [38,39].

While this study provides valuable insight into the potential of salivary CA-125 as a biomarker for OSCC, several limitations must be acknowledged. First, although patients with acute oral infections, including acute periodontitis, were excluded, chronic periodontitis was not systematically assessed. As chronic inflammation may elevate CA-125 levels, this could represent a confounding variable. Future research should incorporate detailed periodontal evaluation or limit inclusion to periodontally healthy participants.

Second, although pregnant, lactating, and menstruating women were excluded due to hormonal influences on CA-125, menopausal status was not documented, potentially introducing variability in the female subgroup. Other unrecorded variables—such as medication use, systemic inflammation, or dietary influences—may also have affected CA-125 levels.

Third, the cross-sectional design and modest sample size limit the generalizability and preclude causal inference. Larger, multicenter studies with robust clinical profiling are needed to validate these preliminary findings.

Fourth, the control group was deemed healthy based on visual oral examination alone, without histopathologic or adjunctive diagnostic screening. As a result, subclinical or anatomically hidden lesions may have gone undetected, potentially leading to the inclusion of undiagnosed OSCC or premalignant cases. This could have resulted in an underestimation of both specificity and negative predictive value. Future studies should employ more rigorous screening protocols, such as autofluorescence, brush biopsy, or imaging, to ensure more accurate control group classification and improve biomarker validation.

Finally, although this study provides valuable insight into the diagnostic potential of salivary CA-125 in OSCC, the current analysis primarily considered the overall TNM clinical staging without isolating the individual contributions of tumor size (T), nodal involvement (N), and distant metastasis (M). Given that salivary biomarkers like CA-125 are locally secreted or transudated from the tumor microenvironment into the oral cavity, the T component—which reflects the size and local invasiveness of the primary lesion—is likely to exhibit the strongest correlation with salivary biomarker levels. In contrast, nodal and distant metastases may not influence salivary composition to the same extent. Therefore, future studies should stratify salivary CA-125 levels according to tumor size (T) to better elucidate its sensitivity in detecting early stage, clinically occult tumors. Such an approach would not only enhance the mechanistic plausibility of CA-125 as a local tumor marker but also refine its clinical applicability in screening protocols aimed at early OSCC detection. The lack of T stage-specific analysis in this study represents a limitation that should be addressed in subsequent investigations.

## 5. Conclusions

This study highlights the potential of salivary CA-125 as a promising non-invasive biomarker for the detection of oral squamous cell carcinoma (OSCC). The significantly elevated levels of salivary CA-125 in OSCC patients, along with its association with tumor differentiation, underscore its diagnostic value. While the findings support previous research, this study uniquely contributes by examining the role of Toombak use and employing an automated immunoassay technique. However, the variability in assay methods, tumor biology, and population-specific factors warrant further investigation. Larger, multicenter studies and longitudinal designs are needed to validate these findings and explore the utility of salivary CA-125 in early diagnosis, disease monitoring, and treatment response.

## Figures and Tables

**Table 1:** Population characteristics of the study participants.

Variable	OSCC Group (n = 50)	Control Group (n = 50)
Number of participants	50	50
Gender		
Males	34 (68%)	34 (68%)
Females	16 (32%)	16 (32%)
Age		
Mean age (years)	59.72 ± 15.35	58.26 ± 14.98
Toombak Use		
Current dipper	19 (38%)	17 (34%)
Ex-dipper	12 (24%)	12 (24%)
Non-dipper	19 (38%)	21 (42%)
Alcohol Consumption		
Current alcohol consumer	3 (6%)	3 (6%)
Ex-alcohol consumer	10 (20%)	10 (20%)
Non-alcohol consumer	37 (74%)	37 (74%)
Smoking		
Current smoker	12 (24%)	12 (24%)
Ex-smoker	11 (22%)	11 (22%)
Non-smoker	27 (54%)	27 (54%)
Chronic Medical Condition		
Presence of chronic medical condition	9 (18%)	8 (16%)
Medically fit	41 (82%)	42 (84%)

**Table 2:** Distribution of OSCC patients according to TNM classification, showing a predominance of advanced-stage tumors.

Stage	Number of Patients (n)	Percentage (%)
Stage I	3	6%
Stage II	3	6%
Stage III	1	2%
Stage IV	43	86%
Total	50	100%

**Table 3:** Salivary CA-125 levels according to histopathological grades of OSCC and post hoc pairwise comparisons (n = 50).

Histopathological Grade	N	Salivary CA-125 Level (U/mL) Median (Range)	Pairwise Comparison	Bonferroni-Corrected *p*-Value	Significance
Well differentiated (G1)	34	336.50 (31.80–1000.00)	G1 vs. G2	1.000	Not significant
Moderately differentiated (G2)	11	278.10 (58.30–864.40)	G1 vs. G3	0.0117	Significant
Poorly differentiated (G3)	5	839.40 (472.40–1340.00)	G2 vs. G3	0.0261	Significant

Overall Kruskal–Wallis *p*-value = 0.014.

**Table 4:** Comparison of the levels of salivary CA-125 according to the site of OSCC (n = 50).

Site of OSCC	N	Level of Salivary CA-125 (Median and Range) U/mL
Multiple sites involved	15	335.70 (31.80–1000.00)
Lips	3	465.30 (96.60–740.00)
Anterior tongue	1	151.80
Floor of the mouth	1	232.20
Buccal mucosa	6	520.30 (306.70–1340.00)
Alveolar ridge	16	403.80 (45.20–956.00)
Hard palate	4	410.20 (155.10–513.00)
Retromolar trigone	2	238.30 (198.50–278.10)
Posterior tongue	2	91.90 (58.30–125.50)
*p*-value		0.326

**Table 5:** Gender influence on salivary CA-125 levels in OSCC patients and healthy controls.

	Groups	Cases Group		Control Group	
Gender		N	Level of Salivary CA-125 (Median and Range) U/mL	N	Level of Salivary CA-125 (Median and Range) U/mL
Males		34	324.20 (31.80–1000.00)	34	188.60 (17.60–1000.00)
Females		16	484.90 (58.30–1340.00)	16	248.15 (76.80–1011.20)
*p*-value		0.134		0.167	

**Table 6:** Influence of snuff dipping on salivary CA-125 level.

	Groups	Cases Group		Control Group	
Toombak Use		N	Level of Salivary CA-125 (Median and Range) U/mL	N	Level of Salivary CA-125 (Median and Range) U/mL
Non-Snuff Dipper		19	355.60 (58.30–1340.00)	21	227.80 (76.80–1011.20)
Snuff Dipper		19	205.70 (31.80–839.40)	17	187.70 (37.00–1000.00)
Ex-Snuff Dipper		12	352.10 (125.50–1000.00)	12	178.25 (17.60–633.90)
*p*-value		0.391		0.628	

**Table 7:** Diagnostic classification of participants based on salivary CA-125 test results. The table shows the number of positive and negative test results among OSCC patients (cases) and healthy individuals (controls), along with group totals.

Test Result	Group		Total
	Cases Group	Control Group	
Positive	24	11	35
Negative	26	39	65
Total	50	50	100

## Data Availability

The data are not publicly accessible because they contain information that might jeopardize the privacy of research participants.

## Abbreviations

The following abbreviations are used in this manuscript:

CA-125	Cancer antigen 125
ELISA	Enzyme-Linked Immunosorbent Assay
FEIA	Fluorescent enzyme immunoassay
OC	Oral cancer
OSCC	Oral squamous cell carcinoma
rpm	Round per minute
U/ml	Units per milliliter
